# The Place of "The Hospital" in Medical Journalism

**Published:** 1896-04-11

**Authors:** 


### The Hospital

An Institutional Journal of

The Medical Sciences and Hospital Administration, with

AN EXTRA NURSING SUPPLEMENT.

Vol. XX.—No. 498.] [April 11, 1896.

### THE PLACE OF "THE HOSPITAL" IN MEDICAL JOURNALISM.

That medicine is highly conservative is one of the most familiar commonplaces of social experience. That in this respect the medicine of the past has been faithfully represented by medical journalism none who know will care to deny. But that modern medicine, albeit conservative to the backbone, is nevertheless as progressive as it is conservative, is one of those obvious and palpable facts which even the most casual of observers can see almost without a look. Journalism played an important part in medicine in the old days when a careful clinicalism was our most scientific method of advance. Now that experimental science has been finally and for ever promoted to the chief place, journalism—the journalism of lucid, direct, convincing, and helpful exposition—is indispensable to the man whose chief opportunities of keeping himself "abreast of the science of the times" are those which his periodical journals afford him.

To be a medical journalist of the highest type is, to our thinking, a great ambition. One thing is very obvious at the present moment, namely, that political journalism is taking the place, not merely of the diplomatic council chamber, but even of Parliament itself. If any reader will consider for a moment the part which a newspaper like the *Times* has played during the recent controversies between England and America, England and Germany, and England and her dependencies in South Africa, he will see that journalism is a power which may be second to none in a political, social, or scientific system.

The Hospital has very precise and definite aims of its own; aims which, so far as appears, no other medical journal has adopted. It aims at presenting the whole of medicine to the whole of the profession. But it attaches its own peculiar significance to "the whole of medicine," and also its own significance to the "whole of the profession." "The whole of medicine" can never be represented by a sick patient in a bed, however many millions he may be multiplied by. Medicine as a whole, the science and art of medicine properly so-called, includes the laboratory work of the physiologist and the pathologist, the complete expositions of the philosophic thinker and systematiser, the general care and management of the sick as carried out, not only in the castle and the cottage, but in hospitals, on the battle-field, and elsewhere; and as conducted, not by the medical attendant and the dispensing chemist only, but by the trained nurse and the hospital administrator also. All this is "medicine"; all this must be taught to the student; all this must be constantly brought to the mind of the practitioner by medical journalism, if this is to be of any service to him. Only as he keeps himself familiar with all these departments of the true philosophy and science of his art can any practitioner be said in any sense to keep himself "abreast of the science of the times." These are the principles which underlie and animate the journalism of The Hospital, and we are striving, as medicine itself is striving, to reach that goal of an ideal perfection which is the only entirely worthy ambition of a true science, a true journal, or a true man.

A little while back we used the expression, "the whole of the profession." To that we attached our own meaning. The true medical profession consists of every honest worker in it. For every honest worker in medicine we desire recognition. As a man's work is recognised so does the man himself become better, and his work also better. It has not been the custom in medicine, either in medical journals or elsewhere, to recognise every honest worker. It has been the custom rather to recognise persons in prominent positions, whether they have been workers or not, and whether they have been honest or not. That is not the way to make our whole profession capable, or serviceable to mankind; neither is it the way to make it honourable in the eyes of mankind. The Hospital has at least wished to do better. And that it has had some success in this we are assured by sympathetic and appreciative letters from medical men of all degrees, and when we find that the general practitioner is encouraged and helped by such efforts as we make on behalf of all branches of the profession, we in turn are encouraged and helped by the thought that we have been helpful to those of our brethren who bear so stern a part of the "heat and burden of the day" as the general practitioner is called upon to bear.

What, then, is the actual position of The Hospital among medical journals? It is that of a newspaper which tries to see medicine frankly and humanly, to see it clearly, and to see it whole. Not only do we desire thus to see medicine, but we desire also to present it in the same clear, sympathetic, and frankly human way to every practitioner of every class who tries honestly to make of medicine a real science and service of man. We do not claim to be in any way honester, wiser, or more brilliant than our journalistic neighbours. But we do claim, and intend to maintain against all comers, the right to teach and to expound, to sympathise with, to stimulate, and to stand loyally by every thing and every man and woman of every class whose business in life is the care and cure of the sick, oe the preservation and improvement of the health, happiness, and working capacity of our country and of mankind.

